# Mechanisms of crosstalk between the oropharyngeal microbiome and human papillomavirus in oropharyngeal carcinogenesis: a mini review

**DOI:** 10.3389/fonc.2024.1425545

**Published:** 2024-08-15

**Authors:** Ryan S. Chung, Stephanie Wong, Dechen Lin, Niels C. Kokot, Uttam K. Sinha, Albert Y. Han

**Affiliations:** Department of Otolaryngology—Head and Neck Surgery, Keck School of Medicine of USC, University of Southern California, Los Angeles, CA, United States

**Keywords:** oropharyngeal cancer, HPV, microbiome, crosstalk, carcinogenesis

## Abstract

Head and neck squamous cell carcinoma (HNSCC) is the sixth most common cancer globally. Notably, human papillomavirus (HPV)-positive oropharyngeal squamous cell carcinoma (OPSCC) is on the rise, accounting for 70% of all OPSCC cases. Persistent high-risk HPV infection is linked to various cancers, but HPV infection alone is not sufficient to cause cancer. Advances in next-generation sequencing have improved our understanding of changes in the human microbiome of cancerous environments. Yet, there remains a dearth of knowledge on the impact of HPV-microbiome crosstalk in HPV-positive OPSCC. In this review, we examine what is known about the oropharyngeal microbiome and the compositional shifts in this microbiome in HPV-positive OPSCC. We also review potential mechanisms of crosstalk between HPV and specific microorganisms. Additional research is needed to understand these interactions and their roles on cancer development and progression.

## Highlights

Head and neck squamous cell carcinoma (HNSCC) is the sixth most common cancer globally. Notably, human papillomavirus (HPV)-positive oropharyngeal squamous cell carcinoma (OPSCC) is on the rise, accounting for 70% of all OPSCC cases. Persistent high-risk HPV infection is linked to various cancers, but HPV infection alone is not sufficient to cause cancer. Additional factors such as chronic inflammation, immune deficiency, and host microbiome alterations may contribute to cancer development. Recent advances in next-generation sequencing have improved our understanding of the role of human microbiome in cancer. This review focuses on the unique features of the oropharyngeal microbiome and its interaction with HPV in carcinogenesis. It highlights the distinct differences between the oral and oropharyngeal microbiomes and the compositional shifts in the microbiome of HPV-positive OPSCC. The review proposes potential mechanisms of HPV-microbiome crosstalk contributing to carcinogenesis, emphasizing the need for further research to understand these interactions and their impact on cancer development and progression.

## Introduction

While the overall incidence of head and neck squamous cell carcinoma (HNSCC) is declining globally, human papillomavirus (HPV)-positive oropharyngeal squamous cell carcinoma (OPSCC) has been increasing, with HPV accounting for 70% of all OPSCC (1–3). HPV is among the most prevalent viral infections worldwide, with nearly all sexually active people being infected with HPV in their lifetime, and half of these being high-risk HPV. Despite this, most people will clear the infection, and only a small minority will have persistent infection (4). It is well-established that persistent high-risk HPV infection is responsible for the development of cervical, anogenital, and oropharyngeal cancers. However, HPV alone is not sufficient to cause cancer (4). Factors such as chronic inflammation, host microbiome alterations, and immune deficiency may be related to pathogenesis, but the mechanisms remain unclear (5).

Recent advances in next generation sequencing have revolutionized our ability to characterize the human microbiome (6). Microbiome studies of different normal and cancer samples have led to improved understanding of associations between microbes and carcinogenesis with potential for risk stratification and prognostication in cancer (7). The oral microbiome harbors over 700 microorganisms (8–10), and disruptions to this microbiome are known to contribute to inflammatory environments with carcinogenic potential (11–14). Firmicutes, Proteobacteria, Bacteroidetes, Fusobacteria, and Actinobacteria have been identified as the most abundant phyla in HNSCC, accounting for > 90% of the oral bacterial community (15, 16). The association between oral dysbiosis and oral squamous cell carcinoma (OSCC) has been well established (15–20). However, the oropharyngeal microbiome and its association with HPV-positive OPSCC are just being uncovered (5). Furthermore, as HPV infection alone is insufficient for cancer development, it is crucial to explore the possibility of HPV-microbial crosstalk and its role in carcinogenesis.

It is important to distinguish the microbiomes of oral cavity and oropharyngeal cancers. The oral cavity microbiome is heavily influenced by environmental factors and subsequent dysbiosis leads to HPV-negative OSCC (19, 21, 22). On the other hand, being primarily HPV driven, OPSCC is an entirely different disease which may be less influenced by environmental factors (23). This review will focus on the unique features of the oropharyngeal microbiome and its interplay with HPV infection in oropharyngeal carcinogenesis. Many reviews discuss the associations between the oral microbiome and OSCC, which will not be discussed. Given the lack of studies on HPV-microbial crosstalk in the context of OPSCC, we also aim to propose potential mechanisms of crosstalk between HPV and specific microbes and highlight areas requiring future study.

## Literature search strategy

We conducted a review of the literature to identify articles addressing the oropharyngeal microbiome, the oropharyngeal microbiome in HPV-positive OPSCC, and HPV-oropharyngeal microbial crosstalk in OPSCC carcinogenesis. This work is not a systematic review; therefore, we did not use PRISMA guidelines. Each query of PubMed’s online repository used the keywords “oropharyngeal microbiome” or “oropharynx [MeSH] microbiota [MeSH].” These keywords were paired with combinations of “human papillomavirus,” “HPV,” “human papillomavirus [MeSH],” and “oropharyngeal neoplasms [MeSH].” Articles were sorted based on relevance and evaluated for content. Exclusion criteria included non-English studies, studies with only pediatric participants, and studies that utilized non-specific oropharyngeal collection methods.

## Microbiome of normal oropharynx and HPV-positive OPSCC

### The normal oropharyngeal microbiome

While there is overlap between the oral and oropharyngeal microbiome, the unique function and histology of the oropharynx lends to differences in how each region interacts with its microbiome. Most studies recognize six major phyla present in the oropharynx, including Firmicutes (44%), Bacteroidetes (20%), Fusobacteria (15%), Proteobacteria (14%), Actinobacteria (6%), and Spirochaetes (0.5%) as comprising over 99% of the microbiome (**Table 1**) (37). The most common genera of bacteria include *Streptococcus* (24%), *Prevotella* (11%), *Fusobacterium* (10%), *Veillonella* (8%), *Neisseria* (5%), and *Actinomyces* (4%). The relative abundance of anaerobic bacteria in the oropharynx is slightly higher than the oral cavity, likely due to the anaerobic niche of the tonsillar crypts (38).

**Table 1:** Summary of the key taxonomic findings in the normal oropharyngeal microbiome based on sampling methods specific to the oropharynx.

Author and Year	Sampling methodology	Sequencing methodology	Key Taxonomic Findings
Siasios et al., 2024 (24)	Oropharyngeal washings	16STM metagenomics	• In COVID-19 positive patients: o **Phyla** (relative abundance %): Firmicutes (41.2%), Proteobacteria (28.4%), Actinobacteria (21.5%), Bacteroidetes (4.6%), Saccharibacteria TM7 (2.8%), Fusobacteria (1.2%), Spirochaetes (0.2%) o **Genera:** *Streptococcus, Veillonella, Peptostreptococcus, Prevotella, Granulicatella, Dolosigranulum, Klebsiella, Actinomyces, Gemella, Haemophilus, Enterococcus, Serratia, Corynebacterium, Tepidiphilus, Acinetobacter, Staphylococcus, Anoxybavillus*
Cui et al., 2024 (25)	Oropharyngeal swabs	16S rRNA	• In healthy controls: o **Phyla:** Firmicutes, Bacteroidota, Proteobacteria, Actinobacteriota and Fusobacteriota accounted for 96.5% of total sequence. o **Genera:** *Prevotella, Streptococcus, Veillonella, Neisseria, Fusobacterium* accounted for 73.45 of total sequence. ▪ **Additional Genera:** *Alloprevotella, Haemophilus, Porphyromonas, Actinomyces, Leptotrichia, Granulicatella, Saccharimonadaceae_TM7x, Rothia, Capnocytophaga, Gemella, Campylobacter*
Oberste et al., 2024 (26)	Swabs from directly visible cancerous tissue or from healthy tissue of the oropharynx and larynx	16S rRNA	• In oropharynx of healthy controls: o **Genera:** *Streptococcus, Haemophilus, Porphyromonas, Prevotellamassila, Peptostreptococcus* (accounted for most of the abundance)
Chung et al., 2023 (27)	Oropharyngeal swabs	16S rRNA	• In nontumor oropharyngeal samples of patients: o **Genera:** *Streptococcus, Neisseria, Haemophilus, Gemella, Porphyromonas, Prevotella*
Lu et al., 2023 (28)	Oropharyngeal swabs	Metatranscriptomic library construction and sequencing	• In healthy volunteers: o **Genera:** *Bacteroides, Actinomyces, Rothia, Prevotella, Dolosigranulum, Veillonella, Mycoplasma, Schaalia, Primorskyibacter* (relative abundance of most common genera). o **Fungal:** *Aspergillus, Clavispora, Daldinia, Malassezia, Pseudogymnoascus, Kwoniella, Fusarium, Komagataella, Saccharomyces, Trametes* (relative abundance of most common fungal profiles)
Wu et al., 2023 (29)	Oropharyngeal swabs	16S rRNA	• In healthy controls: o **Phyla:** Proteobacteria, Bacteroidetes, Firmicutes, Fusobacteria, and Spirochaetes. o **Genera:** *Aspergillus, Capnocytophaga, Corynebacterium, Haemophilus, Leptotrichia, Neisseria, Prevotella, Staphylococcus, Streptococcus*
Xu et al., 2023 (30)	Oropharyngeal swabs	Fungal ITS sequencing	• In healthy controls: o **Phyla:** Ascomycota o **Genera:** *Malassezia, Candida, Zygosaccharomyces, Flammulina, Aspergillus, Rhodotorula, Alternaria, Trichophyton, Talaromyces, Acremonium, Ascochyta*
De Martin et al., 2021 (31)	Tissue collected from tonsils during tonsillectomy and via punch biopsy	16S rRNA	• In patients with OSA: o **Phyla:** Bacteroidetes, Chloroflexi, Cyanobacteria, Epsilonbacteraeota, Firmicutes, Fusobacteria, Patescibacteria, Proteobacteria, Spirochaetes, Synergistetes, Tenericutes, Verrucomicrobia. o Abundance of various phyla did not vary significantly between the tonsillar sampling sites (crypt, epithelium, lymphoid) o **Genera:** *Fusobacterium, Prevotella, Alloprevotella, Treponema, Haemophilus, Veillonella, Prevotella*
Gao et al., 2021 (32)	Oropharyngeal swabs	16S rRNA	• In healthy controls: o **Phyla:** Firmicutes, Bacteroidota, Proteobacteria, and Fusobacteriota. o **Genera:** *Fusobacterium, Haemophilus, Capnocytophaga, Prevotella, Streptococcus, Leptotrichia, Granulicatella, Neisseria, Prevotella, Alloprevotella*
Bach et al., 2021 (33)	Oropharyngeal swabs	Culture-independent metagenomic sequencing	• In healthy participants: o **Phyla:** dominated by Firmicutes, Bacteroidetes, Proteobacteria, Actinobacteria, and Fusobacteria o **Genera:** chiefly *Streptococcus, Veillonella, Prevotella, Neisseria, Actinomyces*
Yang et al., 2019 (34)	Oropharyngeal swabs	16S rRNA	• In Non-OSA participants: o **Genera:** *Prevotella, Neisseria, Veillonella, Fusobacterium, Streptococcus. Prevotella, Neisseria, Veillonella, Fusobacterium, and Streptococcus* made up more than 70% of the bacteria.
Jensen et al., 2013 (35)	Tissue collected from tonsil crypts using sharp surgical spoon. Spoon did not touch outer tonsil surface.	16S rRNA	• In healthy adults: o **Phyla:** Bacteroidetes and Firmicutes were associated with healthy adults. o **Genera:** *Streptococcus, Prevotella, Fusobacterium, Porphyromonas, Neisseria, Parvimonas, Haemophilus, Actinomyces, Rothia, Granulicatella, and Gemella* (core microbiome present in the crypts of human tonsils)
Segata et al., 2012 (36)	Catch-All™ Sample Collection Swabs.	16S rRNA	• In healthy adults: o **Phyla:** Firmicutes, Bacteroidetes, Fusobacteria, Proteobacteria, Actinobacteria, Spirochaetes o **Genera:** *Streptococcus, Prevotella, Fusobacterium, Veillonella, Neisseria, Actinomyces, Leptotrichia, Porphyromonas, Gemella, Haemophilus, Capnocytophaga, Campylobacter, Oribacterium, Granulicatella, Rothia, Actinobacillus, Treponema*

OSA, obstructive sleep apnea.

### The oropharyngeal cancer microbiome

**Figure 1:**
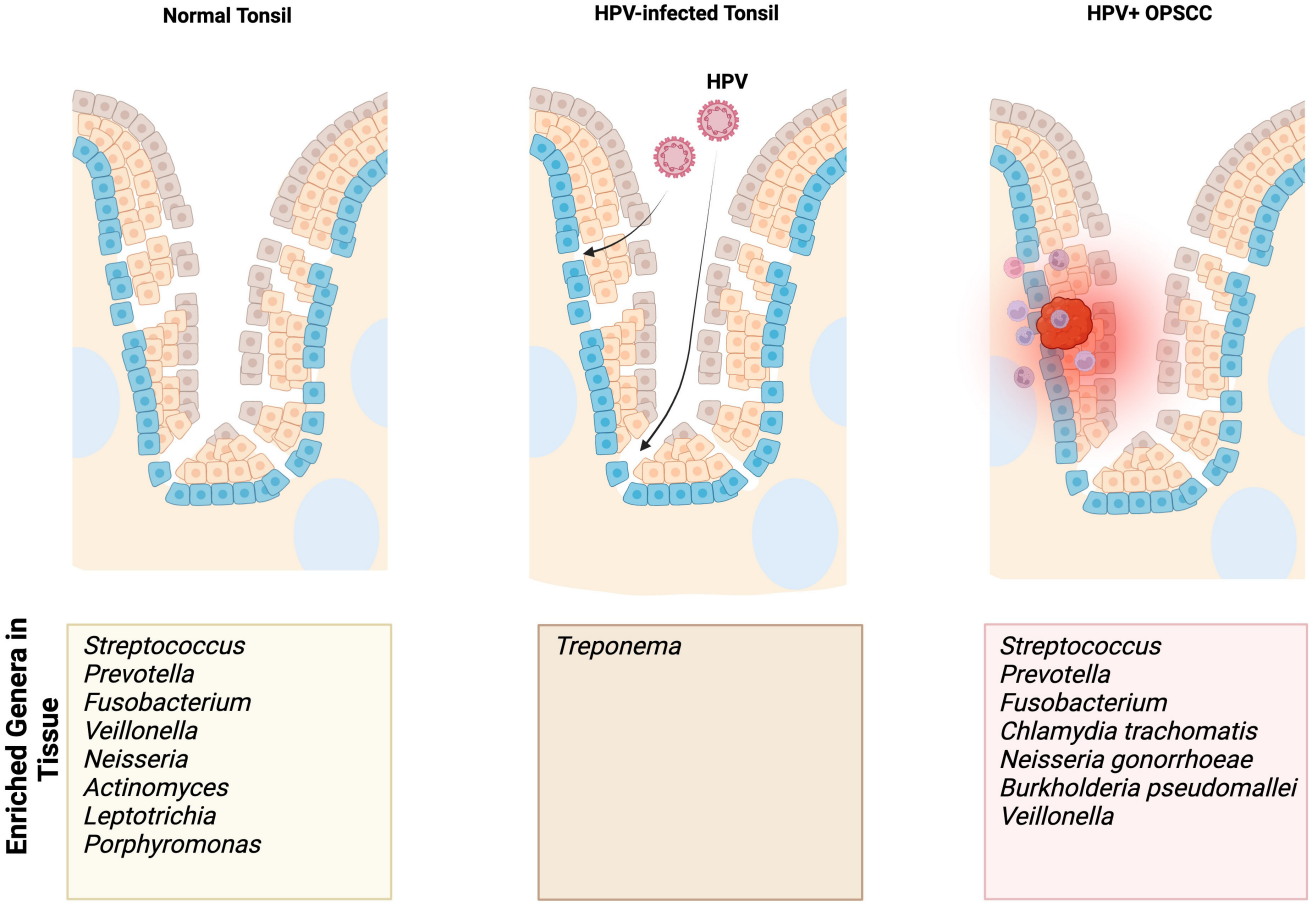
Summary findings of enriched genera/species identified in normal, HPV-infected premalignant, and HPV-positive OPSCC tonsils based on our focused review of the literature. The enriched genera/species outlined in this figure result from studies using sampling methods specific to the tonsils.

Few studies have compared the microbiomes of normal oropharynx and oropharyngeal cancers (**Table 2**). Differences in sample collection ranging from saliva samples or oral washes, which is non-specific to the oropharynx, to oropharyngeal swabs and tissues, which is more specific to the oropharynx, has led to discordance in the literature (38, 44–50). As the normal flora of the oropharynx is dominated by a small number of phyla and genera of bacteria, identifying significant differences between normal and cancer requires careful evaluation using precise methods. Some studies report significant differences in bacteria that account for less than 0.1% of the overall abundance of the whole oropharyngeal microbiome, putting in question the real biological relevance of these differences (38, 44, 49). In this review, we focus on the shifts in relative abundance in the more abundant bacteria of the oropharynx as well as those with evidence of pathogenic roles in association with HPV (**Figure 1**).

**Table 2:** Summary of the key taxonomic findings in the HPV-positive OPSCC microbiome based on sampling methods specific to the oropharynx.

Author and Year	Sampling methodology	Sequencing methodology	Key Taxonomic Findings
Oberste et al., 2024 (26)	Swabs from directly visible cancerous tissue or from healthy tissue of the oropharynx and larynx (larynx samples taken via Kleinsasser tube using an instrument extension)	16S rRNA	• Comparing relative abundance in patients with OPSCC to those without: o **Genera:** Streptococcus (-), Porphyromonas (-),Peptostreptococcus (-), Eubacterium (-), Solobacterium (+), Kingella (-), SR1 (-), Streptobacillus (-), Peptoniphilus, Bacteroides, Alloscardovia (-), Corynebacterium (-), Flavobacteriales (-), Lautropia (-), Flavobacteriaceae (-), Myroides (-), Glutamicibacter (-), Aerococcus (-), Chryseobacterium(-), Paracoccus(-). • Comparing relative abundance in HPV-positive patients to HPV-negative: o **Genera:** *Leptotrichia (+), Staphylococcus (+), Selenemonadaceae (+), Solobacterium (+), Limosilactobacillus (-), Peptoniphilus (-), Paraburkholderia (-), Mobiluncus (+)*. o **Notably, these were only obvious differences, not statistically significant differences**
De Martin et al., 2021 (31)	Tissue collected from tonsils during tonsillectomy and via punch biopsy	16S rRNA	• Comparing relative abundance of HPV p16 positive primary, untreated tonsillar squamous cell carcinoma to OSA controls o **Phyla:** Bacteroidetes (+), Firmicutes (+), Spirochaetes (-), Synergistetes (-). o **Genera:** *Fusobacterium (-), Prevotella (-), Alloprevotella (-), Treponema_2 (-), Streptococcus (+), Haemophilus (no change), Veillonella (+), Prevotella_7 (+).* o **No significant differences in the microbial community composition of tumor-affected vs contralateral tonsils of same patient.** o **Species level analysis of top 10 most predictive in cancer:** ▪ *Porphyromonas NA (-), Fretiacterium NA (-), T. denticola (+), F. periodonticum (+), F. alocis (-), F. nucleatum subsp. vincentii (-), M. micronuciformis (+), P. melaninogenica (+), Streptococcus NA (+), V. atypica (+)*
Rajasekaran et al., 2021 (39)	Tissue specimen from controls (tonsillectomy for OSA) and from cancer (biopsy proven HPV-positive tonsil cancer).	Metagenomics	• Shift towards more gram-negative bacteria in specimens with cancer present • *Burkholderia pseudomallei* was unique to all specimens that contained cancer. • All cancer patient cohorts including the negative nodes shared signatures for *Anaplasma phagocytophilum, Bacillus subtilis, Chlamydia trachomatis, Chlamydophila psittaci, Lactococcus lactis, and Proteus mirabilis.* • Specimens from patients with nodal metastasis contained unique signatures for the gram negative, facultative anaerobe *Shigella dysenteriae*; *Orientia tsutsugamushi* and *Neisseria gonorrhoeae*. • Three species signatures were unique to negative-node primary: o *Leptospira interrogans*, *Sphingomonas wittichii*, and *Coxiella burnetiï* • Five species were unique to positive-node primary: o *Bartonella clarridgeia, Frateuria aurantia, Haemophilus aegyptius, Peptostreptococcus anaerobius, Propionibacterium acnes, and Yersinia pseudotuberculosis*
Oliva et al., 2021 (40)	Oropharyngeal swabs	Metagenomics	• OP swabs in HPV-positive OPSCC: o **Phyla:** Bacteroidetes, Firmicutes, Actinobacteria, Proteobacteria, Fusobacteria o **Genera:** Mostly oropharyngeal anaerobes and facultative anaerobes including *Bacteroides, Prevotella, Veillonella, Streptococcus, Actinomyces, Neisseria.* o Taxonomic composition of oropharyngeal swabs significantly differed across stage III vs stage I-Il patients (p < 0.05). o Four genera were enriched in patients with stage III, including *Fusobacterium (Fusobacterium nucleatum), Gemella (Gemella morbillorum and Gemella haemolysans), Leptotrichia (Leptotrichia hofstadii) and Selenomonas (Selenomonas sputigena and Selenomonas infelix)*
Zakrzewski et al., 2021 (41)	Tumor tissue, Normal tissue within 2 cm of tumor	16S rRNA	• *Treponema* (+) in HPV-positive normal tissue • Changes in genera *Neisseria, Veillonella, Fusobacterium, Prevotella, Porphyromonas* associated with HPV status • *Rothia* (-) in HPV+ tumor tissues
Bahig et al., 2020 (42)	Oropharyngeal swabs of tumor	16S rRNA	• Comparing buccal swab controls to OP swabs of tumor: o **Genera:** *Veillonella (+), Leptotrichia (+)*
Banerjee et al., 2017 (43)	FFPE from mostly HPV-positive OPSCC (HPV16 found in 98% of samples)	PathoChip	• Identifying unique signatures associated with HPV-positive OPSCC when compared to adjacent normal controls (matched) and oral tissue (uvula) o **Genera:** ***Escherichia, Rothia, Peptoniphilus, Brevundimonas, Comamonas, Alcaligenes, Caulobacter, Cardiobacterium, Plesiomonas, Serratia, Edwardsiella, Haemophilus, Frateuria*** **(unique to cancer)**, *Arcanobacterium, Actinomyces, Aeromonas, Bordetella, Aerococcus, Pediococcus, Acinetobacter, Actinobacillus, Veillonella, Mobiluncus, Propionibacterium, Prevotella, Citrobacter, Sphingomonas, Peptostreptococcus, Mycobacterium, Sphingobacterium, Streptococcus* o *Porphyromonas* was unique to normal control o *Campylobacter* and *Streptobacillus* were unique to matched controls o **Fungal:** ***Rhodotorula, Geotrichum, Pneumocystis*** **(unique to cancer)**, *Fonsecaea, Malassezia, Cladosporium, Pleistophora, Absidia, Phialophora, Cladophialophora* o **Parasitic:** ***Hymenolepis, Centrocestus, Trichinella*** **(unique to cancer)**, *Dipylidium, Prosthodendrium, Contracaecum, Toxocara*

HPV, human papillomavirus; OPSCC, oropharyngeal squamous cell carcinoma; OSA, obstructive sleep apnea; FFPE, formalin-fixed, paraffin-embedded.

When comparing the relative abundances of the tonsillar microbiota in patients with OPSCC and OSA controls, De Martin et al. found a relative increase in Firmicutes and Bacteroidetes at the phylum level (48). At the genus level, there was a relatively decreased abundance of *Fusobacterium* and a relatively increased abundance of *Streptococcus* (48). One study found enriched presence of *Chlamydia trachomatis* in HPV-positive tonsil SCC and patients with more advanced disease were colonized with *Neisseria gonorrhoeae* (47).

### Crosstalk between HPV and the oropharyngeal microbiome

Persistent HPV infection in the oropharynx is a known risk factor for the development of OPSCC. While 4% of adults have oral high-risk HPV DNA present, not all develop persistent infection or OPSCC (4, 51–53). Patients with detectable oral HPV (vs non-detectable HPV) and patients with HPV-positive OPSCC (vs HPV-negative OPSCC) have significant shifts in oral microbiome (54–57). Other studies have similarly identified compositional shifts in the vaginal microbiota of pre-malignant lesions and cervical cancer, with mechanistic evidence of HPV-microbial crosstalk contributing to neoplastic progression (58–62). Therefore, HPV-microbial crosstalk may contribute to neoplastic progression in the oropharynx.

After our review of the literature, no study has independently validated the interplay between specific microbes and HPV in OPSCC. Some have reported the oral bacterial signatures of HPV-positive OPSCC, but these are primarily descriptive (9, 17, 63–65). In the subsequent sections, we propose potential mechanisms of crosstalk between HPV and specific microbes identified from our literature search in promoting HPV-related carcinogenesis.

## HPV-Neisseria/Chlamydia: overview

HPV-positive OPSCC patients exhibit tumor microbiome enriched in *Neisseria*, particularly in regionally metastatic disease (**Table 2**) (45–47). Furthermore, one study identified *Neisseria gonorrhoeae* (NG) as the only signature present in patients with nodal disease and *Chlamydia trachomatis* (CT) as a shared signature amongst all HPV-positive OPSCC patient cohorts (**Table 2**) (47). These findings contribute to the growing evidence linking *Neisseria* and *Chlamydia* to persistent HPV infection and HPV-positive cancers of the cervix and oropharynx (66–68).

The carcinogenic potential of the crosstalk between HPV-NG and HPV-CT in the oropharynx may stem from their abilities to enable persistent HPV infection within tissues. Cervicitis is associated with prolonged duration of HPV infection as well as an increased likelihood of high-grade cervical dysplasia (69, 70). Furthermore, high-risk HPV (HR-HPV) and NG co-infection showed an increased risk of atypical squamous cells of undetermined significance (≥ ASC-US) and high-grade squamous intraepithelial lesions (HSIL) (66). HPV-CT co-infection resulted in 5 times higher risk of both ≥ ASC-US and HSIL. A meta-analysis of 22 studies confirmed that HPV-CT co-infection conferred a higher risk of cervical cancer than either alone (71). As NG and CT are primarily GU pathogens, they are thought to be transplanted to the oropharynx during oral sex (59) and subsequently cause chronic subclinical infections, with a predilection to tonsillar tissues where they are difficult to eradicate (72, 73).

### HPV-NG crosstalk: proposed mechanisms

Although both NG and CT contribute to persistence of HPV infection in the oropharynx, their underlying mechanisms may differ. NG disrupts the mucosal barrier by inhibiting epithelial cell renewal and exfoliation. More specifically, NG can engage human carcinoembryonic antigen-related cell adhesion molecules (CEACAMs) present within pharyngeal and urogenital mucosal surfaces by using outer membrane adhesins (74–76). Once engaged, NG blocks detachment of infected epithelial cells from the extracellular matrix (ECM) by triggering the full-length expression of CD105. CD105 is sufficient to inhibit infection-induced detachment, suggesting a mechanism by which NG can disrupt the innate defense mechanism of epithelial exfoliation (77).

*In vitro* studies have demonstrated that NG can also induce DNA damage. Normal vaginal mucosa and cervical adenocarcinoma cell lines infected with NG displayed significantly more DNA strand breaks compared to uninfected controls (78). NG was also shown to induce the cell cycle inhibitors, p21 and p27 (79). Interestingly, NG infected cells can evade DNA damage-induced apoptosis via downregulation of tumor suppressor p53 (78). Through expression of Tfp/pilT, NG can activate the extracellular signal-regulated kinase (ERK) prosurvival pathway resulting in downregulation of proapoptotic proteins Bim and Bad (80). By inhibiting apoptosis in host cells, NG can delay the immune response, triggering a “carrier state” and/or intracellular survival. In HPV-infected epithelium, such a survival mechanism may confer greater immortality in combination with well described oncoproteins HPV E6/E7.

### HPV-CT crosstalk: proposed mechanisms

At a molecular level, CT can also traumatize the epithelium of susceptible mucosal tissues, creating opportunities for microabrasions that promote HPV entry (81). Persistent CT infection in epithelial cells increases the secretion of proinflammatory cytokines IL-8, GROɑ, GM-CSF, and IL-6, which can cause oxidative stress (82). Moreover, the epithelial cells continue to release IL-1ɑ after CT infection, triggering neighboring non-infected epithelial cells to produce additional cytokines, sustaining the inflammatory response and ultimately leading to epithelial tissue damage (82). Of note, HPV-CT co-infected cervical lesions demonstrate significantly upregulated expression of NF-kB compared to non-CT infected cervical lesions. NF-kB is a family of transcription factors that play critical roles in intracellular regulation of immune response and inflammation (83, 84). The upregulated NF-kB pathway caused by HPV-CT co-infection can exacerbate IL-8 mediated epithelial tissue damage, facilitating HPV virion penetrance (85). Furthermore, CT infection has been shown to disrupt N-cadherin-dependent cell-cell junctions in human cervical epithelial cells, resulting in an increase in epithelial paracellular permeability (86). This may explain how CT can increase the risk of HPV acquisition and persistence and how HPV-CT co-infection can increase risk of carcinogenesis (87, 88).

Beyond epithelial disruption, CT can also disturb the immune response necessary to clear HPV, leading to persistent infection. CT can cause a shift in the immune landscape from a cell-mediated response to a humoral response due to its chronic infection in mucosal tissues (83, 89, 90). As cell-mediated immune responses are critical for clearance of HPV infections, a shift to a humoral response creates an environment where HPV infection can thrive (91). Indeed, a cohort study of Swedish women showed that a self-reported history of CT infection was the most significant risk factor for presence of HPV DNA in the blood (92). Furthermore, the CT outer membrane pore protein OmpA can inhibit apoptosis in infected host cells by targeting the pro-apoptotic proteins Bax and Bak. Similar to HPV-NG crosstalk, inhibition of apoptosis may compound the anti-apoptotic effect of HPV E6/E7 and is a hallmark of carcinogenesis (93).

Chronic CT infection can also promote carcinogenesis via DNA damage caused by reactive oxygen species (94). One study used an HPV16 infected ectocervix organoid and CT co-culture system to model HPV-CT crosstalk (61). Using this model, they found that CT impedes HPV-induced mechanisms that maintain genome integrity, including mismatch repair, creating a genotoxic effect in host cells. Furthermore, HPV16 E6/E7 integration in the host genome slows the CT life cycle and induces persistent infection. This study demonstrates the potential complexity of this bidirectional relationship between CT and HPV in cancer development and progression (61).

### Summary of HPV-NG/CT crosstalk

The crosstalk between HPV-NG and HPV-CT may contribute to carcinogenesis in the oropharynx by disrupting the normal mucosal barrier and facilitating entry of HPV virions, delaying immune response, and promoting genotoxicity. Additional research is required to evaluate the impact of HPV-NG and HPV-CT co-infection in oropharyngeal models.

### HPV-Fusobacterium: overview and proposed mechanisms

*Fusobacterium spp* are well established periodontal pathogens (95). Of these, *Fusobacterium nucleatum* (FN) is well known for its role in OSCC development (96–98). FN colonization increases risk of OSCC by augmenting IL-6-STAT3 signaling pathways, promoting tumor growth and invasiveness (15, 99). However, studies on the oropharyngeal microbiome suggest the contrary: HPV-positive OPSCC exhibits a reduction of *Fusobacterium spp* compared to the normal oropharynx (48). As with NG and CT, such differences can be partially explained by differences in experimental methods: lack of stratification of HPV-positive to HPV-negative, sampling methods, and sequencing methodology (100).

Although less prominent in HPV-positive OPSCC compared to otherwise healthy adults, progressive over-representation of FN has been characterized with stage III HPV-positive OPSCC patients, showing a significantly higher relative abundance of FN compared to earlier stages. This correlation between FN representation and stage is consistent with that of OSCC and gastrointestinal squamous cell carcinoma studies (46, 98, 101–103). Furthermore, some have proposed that patients with stage III HPV-positive OPSCC are at a higher risk of recurrence despite definitive concurrent chemoradiation due to differences in FN enrichment (104). Ultimately, the etiology explaining these associations remain unclear. HPV-FN co-infection in the tonsillar tissues of susceptible patients can be a contributing factor.

**Figure 2:**
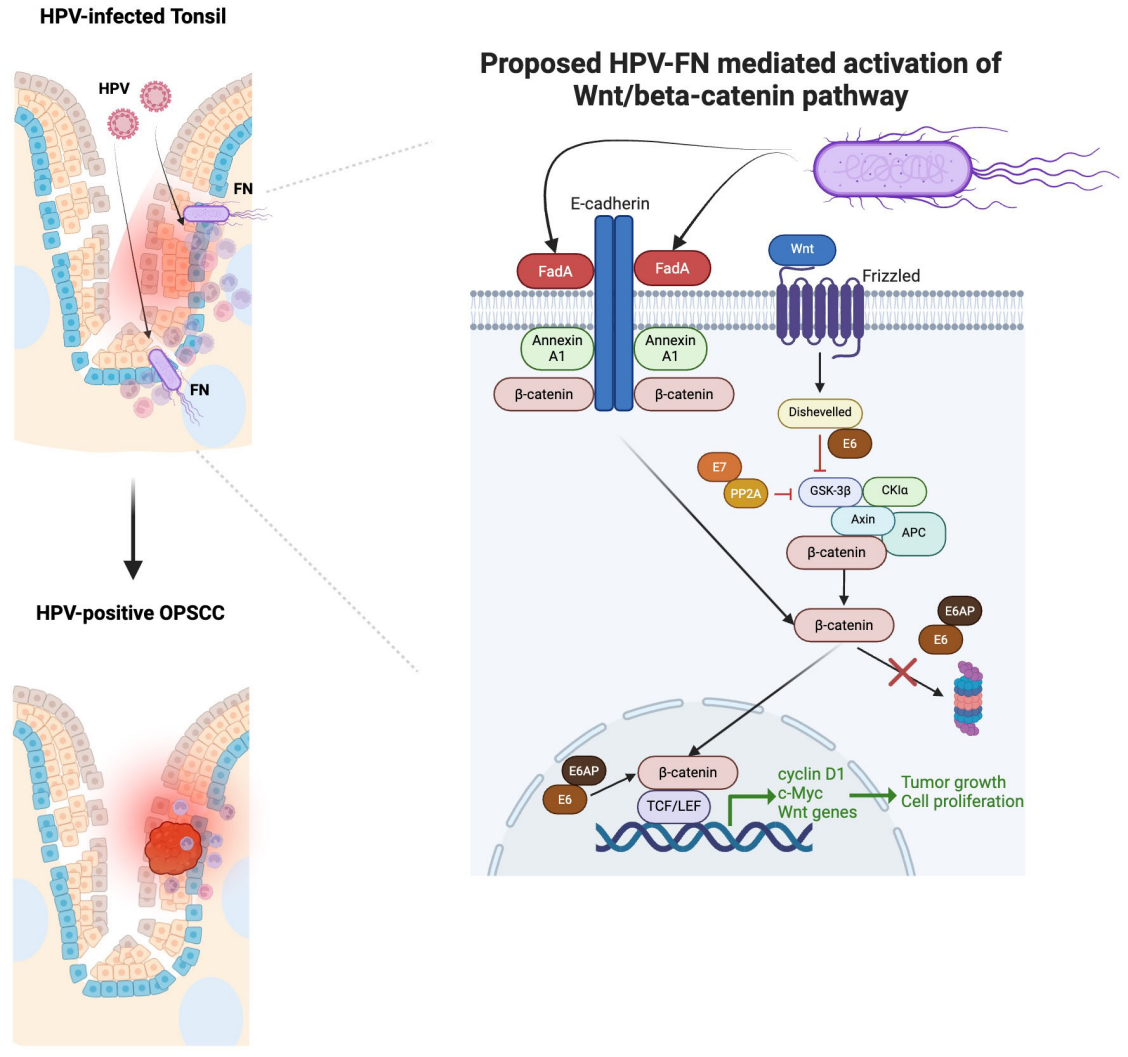
Proposed multiplicative effect HPV-*Fusobacterium nucleatum* (FN) crosstalk in contributing to HPV-positive OPSCC based on our focused review of the literature. FadA binding enhances when Annexin A1 level increases (105). Annexin A1 is significantly elevated in the margins of HPV-positive OPSCC compared to HPV-negative OPSCC (108). FadA can interact with E-cadherin of epithelial cells to stimulate the Wnt/β-catenin pathway (105). As a result, β-catenin can activate the expression of cyclin D1, c-Myc, and Wnt signaling genes, which promote cell proliferation and tumor growth. HR-HPV oncoproteins E6/E7 can also activate the canonical Wnt/β-catenin pathway, which further contribute to the onset, progression, and maintenance of transformed cells (106). E7 binds to PP2A in the structural and catalytic domain to contribute to the inhibition of GSK3β activation (106). The E6/E6AP complex can stabilize β-catenin, avoiding its proteasomal degradation and promoting its nuclear translocation. The binding of E6 to Dishevelled can disrupt the β-catenin degradation complex.

The potential mechanisms by which FN contributes to cancerous growth have been studied in the colon and oral cavity. FadA, a virulence factor expressed by FN, is a cell surface adhesion protein involved in the attachment and invasion of epithelial cells. It can interact with E-cadherin of epithelial cells to stimulate the Wnt/β-catenin pathway (105). As a result, β-catenin can activate the expression of cyclin D1, c-Myc, and Wnt signaling genes, which promote cell proliferation and tumor growth. HR-HPV oncoproteins also activate the canonical Wnt/β-catenin pathway, which further contribute to the onset, progression, and maintenance of transformed cells (106). Studies in cervical cancer have highlighted the role of Wnt signaling as the “second hit,” responsible for transforming HPV-immortalized cells (107). Therefore, HPV-FN crosstalk may result in a multiplicative effect on the growth of HPV-positive cancers (**Figure 2**).

HPV-FN crosstalk may also alter mucosal integrity, predisposing to HPV infection and OPSCC progression. FN produces matrix metalloproteinases, which are capable of degrading components of the extracellular matrix (ECM) (109). FN can also promote the epithelial-mesenchymal transition (EMT), predisposing cells to malignant transformation. One study in oral epithelial cells demonstrated that FN infection can trigger EMT in both normal and cancerous cells via the lncRNA MIR4435–2HG/miR-296–5p/Akt2/SNAIl signaling pathway, a process that also downregulates E-cadherin (110), thereby breaking down the epithelial barrier and allowing HPV infection and malignant transformation (111). Additional research must explore the mechanisms by which HPV-FN crosstalk can foster tumor growth, progression, and clinical outcomes.

### HPV-Streptococcus: overview and proposed mechanism

Oropharyngeal microbiome studies have also revealed that HPV-positive OPSCC patients demonstrate enriched signatures of the genus *Streptococcus* (38, 46, 48). *Streptococcus* has been heavily associated with both periodontal inflammation as well as OSCC, and is one of the most common microbes in the oral cavity and oropharynx (11, 15, 16, 112). Furthermore, one study of salivary samples from patients with OPSCC revealed that the relative abundance of *Streptococcus* could be used to differentiate tumors from control samples (17). As *Streptococcus spp* are commensal to the oral cavity and oropharynx, environmental insults that trigger dysbiosis may allow for opportunistic organisms to colonize (113). *Streptococcus mutans* (SM), a viridans group streptococcus (VGS), represents one of these species. SM has been shown to induce IL-6 expression in human and mouse oral cancer cell lines, representing a specific mechanism for its potential carcinogenicity (114). As with the other microbes mentioned, it is uncertain whether the changes observed in streptococci are the cause or consequence of cancer.

One study demonstrated that *Streptococcus* may promote HPV16 entry into basal keratinocytes by supplying a furin-like peptidase (115). HPV16 infection is initiated by binding to receptor heparan sulfate proteoglycans (HSPGs) on the exposed basement membrane (81, 115, 116). Following binding to HSPGs, the L2 cleavage site is exposed and subsequently cleaved by furin, facilitating HPV16 entry into tissues (116). Unlike HSPGs, the expression of furin varies based on tissues (117). In the human oropharyngeal epithelium, furin expression is low in the basal layers where HPV16 typically initiates infections (118). One study revealed that PepO, the furin-like peptidase of *S. gordonii*, promoted HPV16 infection (119). Altogether, this suggests that HPV-*Streptococcus* crosstalk may allow for better penetration of HPV into the basal keratinocytes, where it can proliferate.

## Conclusions and future directions

The intricate composition of the oropharyngeal microbiome is only beginning to be understood. Previous studies have focused on saliva due to ease of access (16, 65, 120). Although these findings have been used as a mirror of the oropharyngeal microbiome, targeted collection methods have shown unique signatures of bacteria and other microbes in this region (37, 47, 48). Traditional periodontal pathogens found in OSCC (*Porphyromonas gingivalis* and *Treponema denticolum*) did not present as key players (19, 121). Instead, FN and SM provide pro-inflammatory environments, in conjunction with chronic HPV infection, that may increase propensity for the development of cancer (46, 48). High quality mechanistic studies have yet to provide a comprehensive landscape of these interactions critical for the development of novel therapy.

We propose that HPV-microbial crosstalk impacts patient outcomes at multiple levels. First, it facilitates HPV virion entry to the basal keratinocytes and fosters an immune environment that also allows for HPV persistence. Furthermore, as demonstrated in other solid tumors, direct and secreted microbial features may confer treatment resistance in advanced stages of HPV-positive OPSCC (101, 103). Key microbial signatures of HPV-positive OPSCC may provide useful biomarkers for deintensification of patients.

Novel reductionist approaches utilizing tissue-microbe co-culture may be one way to model microbial effects on HPV-associated carcinogenesis (61). These studies would also benefit from including fungi, proteus, and bacteriophages, which occupy their own niche roles in the microbiome. In cervical cancer, specific fungal communities (*Candida*, *Malassezia*, and *Sporidiobolaceae*) were identified as significantly associated with HR-HPV and premalignant cervical lesions (122). We believe similar associations occur in the oropharynx, triggering carcinogenesis in the HPV-infected tonsil. As the incidence of HPV-positive OPSCC begins to exceed that of cervical cancer, it is necessary to understand the functional relationships between microbes and HPV to characterize oropharyngeal premalignant lesions and enhance our understanding of HPV-related carcinogenesis (123).
